# Peripheral Blood Eosinophilia Is Associated with Poor Outcome Post-Lung Transplantation

**DOI:** 10.3390/cells9112516

**Published:** 2020-11-20

**Authors:** Janne Kaes, Elise Van der Borght, Arno Vanstapel, Anke Van Herck, Annelore Sacreas, Tobias Heigl, Bart M. Vanaudenaerde, Laurent Godinas, Dirk E. Van Raemdonck, Laurens J. Ceulemans, Arne P. Neyrinck, Robin Vos, Geert M. Verleden, Stijn E. Verleden, Group the Leuven Lung Transplant

**Affiliations:** 1 Laboratory of Respiratory Diseases and Thoracic Surgery (BREATHE), Department of Chronic Diseases and Metabolism, KU Leuven, B-3000 Leuven, Belgium; janne.kaes@kuleuven.be (J.K.); elise.vanderborght@student.kuleuven.be (E.V.d.B.); arno.vanstapel@kuleuven.be (A.V.); anke.vanherck@kuleuven.be (A.V.H.); annelore.sacreas@kuleuven.be (A.S.); tobias.heigl@kuleuven.be (T.H.); bart.vanaudenaerde@kuleuven.be (B.M.V.); dirk.vanraemdonck@uzleuven.be (D.E.V.R.); laurens.ceulemans@uzleuven.be (L.J.C.); robin.vos@uzleuven.be (R.V.); geert.verleden@uzleuven.be (G.M.V.); 2 Department of Pathology, UH Leuven, B-3000 Leuven, Belgium; 3 Department of Respiratory Diseases, Lung Transplant Unit, UH Leuven, B-3000 Leuven, Belgium; laurent.godinas@uzleuven.be; 4 Department of Thoracic Surgery, UH Leuven, B-3000 Leuven, Belgium; 5 Laboratory of Anesthesiology and Algology, Department of Cardiovascular Sciences, KU Leuven, B-3000 Leuven, Belgium; arne.neyrinck@uzleuven.be

**Keywords:** lung transplantation, eosinophilia, chronic lung allograft dysfunction

## Abstract

Eosinophils play a role in many chronic lung diseases. In lung transplantation (LTx), increased eosinophils in bronchoalveolar lavage (BAL) was associated with worse outcomes. However, the effect of peripheral blood eosinophilia after LTx has not been investigated thoroughly. A retrospective study was performed including all LTx patients between 2011–2016. Chronic lung allograft dysfunction (CLAD)-free and graft survival were compared between patients with high and low blood eosinophils using an 8% threshold ever during follow-up. A total of 102 patients (27.1%) had high blood eosinophils (≥8%) (45 before CLAD and 17 after, 40 had no CLAD) and 274 (72.9%) had low eosinophils (<8%). Patients with high blood eosinophils demonstrated worse graft survival (*p* = 0.0001) and CLAD-free survival (*p* = 0.003) compared to low eosinophils. Patients with both high blood and high BAL (≥2%) eosinophils ever during follow-up had the worst outcomes. Within the high blood eosinophil group, 23.5% had RAS compared to 3% in the group with low eosinophils (*p* < 0.0001). After multivariate analysis, the association between high blood eosinophils and graft and CLAD-free survival remained significant (*p* = 0.036, *p* = 0.013) independent of high BAL eosinophils and infection at peak blood eosinophilia, among others. LTx recipients with ever ≥8% blood eosinophils demonstrate inferior graft and CLAD-free survival, specifically RAS, which requires further prospective research.

## 1. Introduction

Lung transplantation (LTx) is a life-saving treatment for selected patients with end-stage pulmonary diseases, such as emphysema, cystic fibrosis and pulmonary fibrosis. Short-term survival post-LTx improved significantly due to changes in donor procedures (selection and preservation), perioperative management and improved handling of post-operative complications. Long-term outcomes, however, are still lagging behind compared to the survival following other solid organ transplants. Complications hampering long-term survival after LTx are chronic lung allograft dysfunction (CLAD), cancer and side effects of the life-long necessary immunosuppression, e.g., infections and renal insufficiency [1]. CLAD is the major drawback, occurring in approximately 50% within five years after transplantation [2].

The main methods to monitor the pulmonary condition post-LTx are spirometry, transbronchial biopsies, chest imaging and analysis of bronchoalveolar lavage (BAL) samples. Moreover, it is important to identify and avoid risk factors that contribute to the development of CLAD or other complications. Many risk factors have been described, e.g., acute rejection, lymphocytic bronchiolitis, BAL neutrophilia, infection/colonization, primary graft dysfunction [3], presence of persistent donor-specific antibodies (DSAs) [4], gastroesophageal reflux [5] and air pollution [6].

In 2014, our group showed that BAL eosinophilia of ≥2% is associated with worse CLAD-free and overall survival post-LTx [7]. Eosinophils are granulocytes that belong to the innate immune system and are considered destructive and cytotoxic in asthma [8]. The exact role of eosinophils after LTx is largely unknown. In 1998, Trull et al. were the first to demonstrate that an increased peripheral blood eosinophil count was an early marker of acute pulmonary rejection [9]. Furthermore, blood eosinophils are systemically increased during acute rejection episodes of the lung allograft [10]. During pulmonary infections (bacteria, viruses, fungus), eosinophils appear to play an important role in the host defense through a wide range of mechanisms [11]. However, no comprehensive study has investigated the prospective value of peripheral blood eosinophilia post-LTx, which is the aim of our current study.

## 2. Materials and Methods

### 2.1. Patient Inclusion

All patients transplanted between January 2011 and December 2016 at the University Hospitals Leuven were included in this retrospective study (*n* = 397). Patient follow-up was censored on 29th of May 2019. Patients with a follow-up of fewer than six months were excluded (*n* = 21). Blood and BAL samples taken before post-operative day 90 were not taken into account due to interference with donor characteristics, as described previously [7]. Retransplantation was considered as a separate event. Peripheral blood total and differential cell counts were routinely performed at the University Hospital laboratory. For each included patient, every peripheral blood eosinophil percentage during their post-LTx follow-up was checked in the electronic patient files and all values were recorded. The study was approved by the local Ethics Committee (MP010035) and all patients provided written informed consent to participate in scientific studies at time of listing for transplantation.

### 2.2. Definitions

CLAD, BOS and RAS were identified according to the recent guidelines from the International Society for Heart and Lung Transplantation (ISHLT) using a combination of spirometry, body plethysmography and chest CT scan [12,13]. In case patients did not develop CLAD, the date of their last patient contact or death was used for censoring. The threshold for high blood eosinophils was based on the median percentage of the peak blood eosinophils of deceased patients (7.4%; 3.9–12). Subsequently, a receiver operating characteristic (ROC) analysis was performed, resulting in an optimal cut-off of >7.85% to predict graft survival (sensitivity 47.89%; specificity 76.72%). Consequently, a threshold of 8% was used for further analyses. Patients were divided into two groups according to the defined threshold: one group with “high blood eosinophils” (≥8%) and a group with “low blood eosinophils” (<8%) during follow-up. If patients developed high blood eosinophils after CLAD diagnosis, they were considered as “low blood eosinophils” for CLAD analysis and as “high blood eosinophils” for graft survival analysis. Graft survival was a combined end-point of death or retransplantation. Separate episodes of high blood eosinophils (≥8%) in the same patient were defined as two elevated measurements with at least three months in between. High BAL eosinophils was defined as ≥2% eosinophils present in BAL [7]. BAL was performed as previously described [7]. All patients included in this study received a standardized immunosuppressive regimen, consisting of induction therapy with anti-thymocyte globulin (rATG) (3 mg/kg/d for three days) and subsequent conventional triple-drug immunosuppressive maintenance therapy consisting of methylprednisolone, a cytostatic agent (mycophenolate mofetil or azathioprine) and a calcineurin inhibitor (tacrolimus or cyclosporine). Transbronchial biopsies were scored for acute rejection (Grade A, severe AR ≥ A2) and lymphocytic bronchiolitis (Grade B, severe LB ≥ B2) according to ISHLT guidelines [14]. Total number and mean (sum per patient, divided by the number of biopsies taken during follow-up) AR and LB scores were reported. DSAs were determined as previously reported [4]. Infection was defined as a pulmonary infection of any kind (i.e., respiratory symptoms such as cough, increased sputum production, shortness of breath in combination with fever and/or elevated C-reactive protein (CRP) and/or alterations on chest X-ray), requiring the use of antibiotics, antivirals or antifungals. The use of meropenem was further investigated as this is a potential inducer of peripheral blood eosinophilia [15].

### 2.3. Statistical Analyses

GraphPad Prism version 8.3.0 (GraphPad Software, San Diego, CA, USA) was used for statistical analyses. Threshold for “high blood eosinophils” was validated using ROC curve analysis of each patient’s individual highest blood eosinophil percentage after LTx to predict graft survival. Kaplan–Meier analysis with log-rank test was used to compare overall graft survival and freedom from CLAD after LTx. Contingency tables were analyzed using Fisher’s exact test or chi-square test, where appropriate. Multivariate analyses were performed in SPSS version 26 (SPSS Statistics, IBM, New York, NY, USA) using the Cox proportional hazard model after univariate analyses of all clinically relevant binary covariates. Data are presented as percentages or as total values, median and interquartile range where appropriate. A *p*-value of <0.05 was considered significant.

## 3. Results

During the inclusion period, 397 LTx were performed, of which 21 (5.3%) patients did not reach the required follow-up of six months and were consequently excluded.

### 3.1. Patient Characteristics

Among the 376 included patients, 274 patients (72.9%) had low blood eosinophils (<8%), whereas 102 patients (27.1%) experienced one or more episodes of high blood eosinophils (≥8%) during their post-transplant follow-up. There were significantly more females in the high eosinophil group (62.7%) compared to the low eosinophil group (44.5%) (*p* = 0.002). In the high eosinophil group, there were also more patients with cystic fibrosis and/or bronchiectasis compared to the group with low eosinophils (*p* = 0.014). During their entire post-transplant follow-up, more biopsies were taken in the high eosinophil group (7 (6–8) vs. 6 (6–7); *p* = 0.0047). Within these transbronchial biopsies, significantly more episodes of lymphocytic bronchiolitis (*p* = 0.012) and more severe lymphocytic bronchiolitis (≥B2) (*p* = 0.031) were observed in the high eosinophil group. No significant difference was observed for acute perivascular rejection episodes neither for severe acute rejection episodes (*p* = 0.21 and *p* = 0.11). Donor-specific antibodies (DSAs) were found more frequently in the group with high blood eosinophils compared to low blood eosinophils (14.7% vs. 6.6%; *p* = 0.022). Detailed patient characteristics are given in Table 1.

### 3.2. High Peripheral Blood Eosinophils and Graft Survival

At the end of the study, 33/102 patients (32.4%) died or underwent retransplantation in the high blood eosinophil group (median follow-up time: 4.5 year (3.0–6.5)) in contrast with only 38/274 (13.9%) in low blood eosinophil group (median follow-up time: 4.7 year (3.2–6.6)) (Table 1). In the high eosinophil group, the major reason for death or retransplantation was respiratory insufficiency due to CLAD (63.6%), compared to 31.6% deaths due to CLAD in the low group. Infection was the cause of death in 28.9% of the patients in the low group whereas only 9.1% died of an infection in the high eosinophil group. The remaining deaths were multifactorial or other, e.g., cancer, cardiac problems (Table 1). The 33 patients in the high eosinophil group died in a median time of 7.8 months (5.2–21.5) after the first episode of high blood eosinophils.

**Figure 1:**
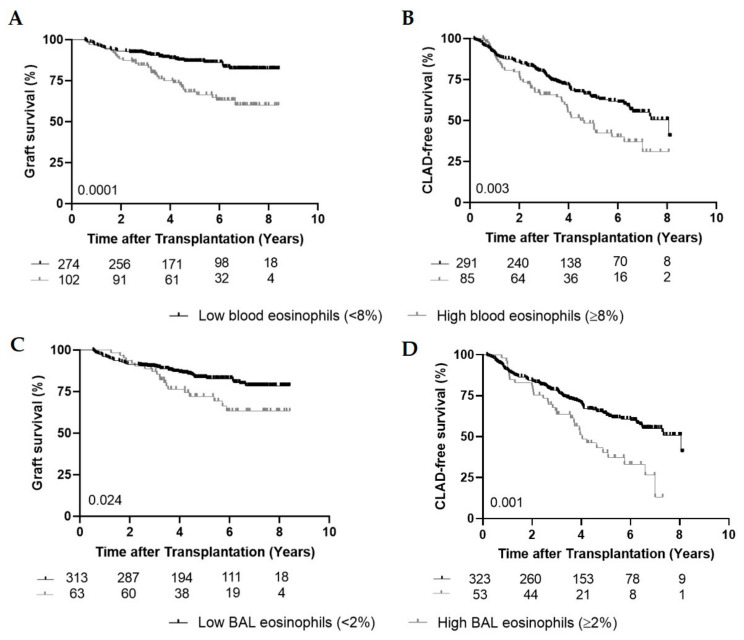
Graft and CLAD-free survival. (**A**), Kaplan–Meier curve comparing overall graft survival (%) between the low blood eosinophil group (<8%) (*n* = 274) and the high blood eosinophil group (≥8%) (*n* = 102) (*p* = 0.0001). (**B**), Kaplan–Meier curve comparing CLAD-free survival (%) between the low blood eosinophil group (<8%) (*n* = 291) and the high blood eosinophil group (≥8%) (*n* = 85) (*p* = 0.003). (**C**), Kaplan–Meier curve comparing graft survival (%) between the low bronchoalveolar lavage (BAL) eosinophil group (<2%) (*n* = 313) and the high BAL eosinophil group (≥2%) (*n* = 63) (*p* = 0.024). (**D**), Kaplan–Meier curve comparing CLAD-free survival (%) between the low BAL eosinophil group (<2%) (*n* = 323) and the high BAL eosinophil group (≥2%) (*n* = 53) (*p* = 0.001).

Graft survival was significantly shorter in the high eosinophil group compared to the low group (five-year survival of 66.5% compared to 86.6%; HR = 2.44; CI: 1.53–3.89) (*p* = 0.0001) (Figure 1A). Fifty-four patients (53%) experienced one episode of high blood eosinophils, whereas in 48 patients (47%) multiple episodes (≥2 with at least three months in between) were found (maximum of 14 episodes). There was no difference in graft survival between patients with multiple episodes of high blood eosinophils and those with only one episode (*p* = 0.64), but in both groups, the survival was significantly inferior compared to patients with low blood eosinophils (*p* = 0.006 and *p* < 0.0001).

### 3.3. High Peripheral Blood Eosinophils and CLAD-Free Survival

In the high blood eosinophil group (*n* = 102), 62 patients (60.8%) were diagnosed with CLAD, of which 17 patients (27.4%) developed CLAD before their first episode of high blood eosinophils and 45 patients (72.6%) developed CLAD after the first episode of high blood eosinophils (median time: 5.5 months (1.9–21.8)). CLAD was diagnosed in only 30.3% (*n* = 83) in the group with low eosinophils compared to the 60.8% (*n* = 62) in the high group (*p* < 0.0001) (Table 2). There were significantly more diagnoses of RAS in the high eosinophil group (23.5%) compared to the group with low eosinophils (3%) (*p* < 0.0001). Concerning BOS, no difference was observed between both groups (*p* = 0.08) (Table 2).

To assess the effect of high peripheral eosinophils on CLAD-free survival, we identified 85 patients (22.6%) with high blood eosinophils (≥8%), whereas 291 patients (77.4%) had blood eosinophils below the threshold of 8% (17 patients only developed high blood eosinophils after CLAD diagnosis and are therefore belonging to the low eosinophil group for the CLAD-free analysis). CLAD-free survival was shorter in the high eosinophil group compared to the group with low eosinophils (HR = 1.69; CI: 1.19–2.41; *p* = 0.003) (Figure 1B). Having multiple episodes of high blood eosinophils did not affect CLAD-free survival compared to having one episode (*p* = 0.31).

### 3.4. Role of Infection and Meropenem at Peak Peripheral Blood Eosinophilia

During peak blood eosinophilia, 48% had an active infection in the high blood eosinophil group (≥8%) compared to only 25% in the low blood eosinophil group (<8%) (*p* < 0.0001). The infections were in both groups mainly of pulmonary origin (69.4% vs. 65.2%). In the high blood eosinophil group, most cases were attributable to multiple possible pathogens (35.3%), while in the group with low blood eosinophils, most pulmonary infections were caused by a bacterial pathogen (44.4%). The remaining infections were extrapulmonary (8.2% vs. 10.2%) or of unknown origin (22.4% vs. 24.6%) (Table A1 in Appendix A).

Associated with infection is the administration of meropenem, 32.4% of the patients received meropenem in the high blood eosinophil group at the moment of peak eosinophilia compared to 6.9% in the group with low blood eosinophils (*p* < 0.0001).

### 3.5. High BAL Eosinophils, Graft and CLAD-Free Survival

Of all patients, 63 (16.8%) experienced high BAL eosinophils (≥2%) during their post-transplant follow-up. Of these 63 patients, 42 patients (66.7%) had CLAD, of which 15 patients (35.7%) developed CLAD before high BAL eosinophils and 27 (64.3%) developed CLAD after the first high BAL eosinophil episode (median time: 1.07 year (0.046–3.01)) (Table 2). Univariate analysis demonstrated that graft and CLAD-free survival were significantly shorter in the group with high BAL eosinophils compared to low BAL eosinophils (HR = 1.82; CI: 1.08–3.08; *p* = 0.024 and HR = 1.90; CI: 1.28–2.82; *p* = 0.001) (Figure 1C,D).

### 3.6. High Peripheral Blood Eosinophils and High BAL Eosinophils

Of the 63 patients with high BAL eosinophils, there were 27 (42.9%) with low peripheral blood eosinophils and 36 (57.1%) with high peripheral blood eosinophils. In 28 (77.8%) of these 36 patients, high blood eosinophils occurred 4.4 months (1.1–10.3) before high BAL eosinophils, while eight patients (22.2%) first experienced high BAL eosinophils and only 2.6 years (3.9–0.59) later, they developed high blood eosinophils.

**Figure 2:**
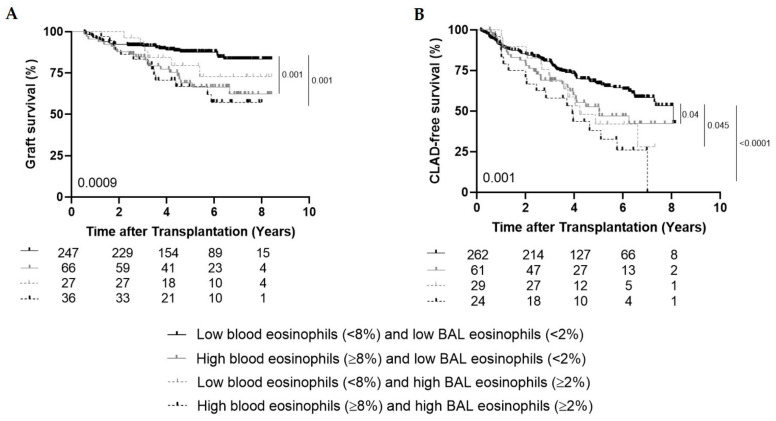
High blood (≥8%) and high BAL (≥2%) eosinophils. (**A**), Kaplan–Meier curve comparing graft survival (%) between the group with low blood and low BAL eosinophils (*n* = 247), the group with high blood and low BAL eosinophils (*n* = 66), the group with low blood and high BAL eosinophils (*n* = 27) and the group with both high blood and high BAL eosinophils (*n* = 36) (*p* = 0.0009). (**B**), Kaplan–Meier curve comparing CLAD-free survival (%) between the group with low blood and low BAL eosinophils (*n* = 262), the group with high blood and low BAL eosinophils (*n* = 61), the group with low blood and high BAL eosinophils (*n* = 29) and the group with both high blood and high BAL eosinophils (*n* = 24) (*p* = 0.001).

Graft survival was shorter in patients with high blood (≥8%) and high BAL (≥2%) eosinophils (*n* = 36) ever during follow-up and in patients with high blood eosinophils and low BAL eosinophils (*n* = 66) compared to patients with low blood and low BAL eosinophils (*n* = 247) (*p* = 0.001 and *p* = 0.001 respectively) (Figure 2A). CLAD-free survival was significantly shorter in patients with both high blood and high BAL eosinophils (*n* = 24), in patients with high blood eosinophils and low BAL eosinophils (*n* = 61) and in patients with low blood and high BAL eosinophils (*n* = 29) compared to patients with both low blood and low BAL eosinophils (*n* = 262) (*p* < 0.0001; *p* = 0.04; *p* = 0.045 respectively) (Figure 2B).

### 3.7. Histological Findings in Explant Lungs of Retransplantations

There were 10 retransplantations within our cohort. Within the lungs explanted from patients with low blood eosinophilia (*n* = 5), fibrous obliteration of the airway was found in all cases (100%), while diffuse parenchymal fibrosis was found in two cases (40%) and pleural thickening was observed in two cases (40%). Within the lungs from patients with high blood eosinophilia (*n* = 5), four showed obliteration of the airways (80%), four showed parenchymal fibrosis (80%) and two showed pleural thickening (40%).

### 3.8. Multivariate Analyses

Multivariate analyses included covariates which were significantly associated with either graft survival or CLAD-free survival in univariate analysis (age, underlying disease, acute rejection, DSAs, infection at peak blood eosinophilia and meropenem at peak blood eosinophilia), covariates previously associated with high peripheral blood eosinophils in this study (gender and lymphocytic bronchiolitis), and both high blood and high BAL eosinophils. Multivariate analysis confirmed the association between high blood eosinophils and worse graft survival (HR = 1.81; CI: 1.04–3.16; *p* = 0.036) and shorter CLAD-free survival (HR = 1.67; CI: 1.11–2.50; *p* = 0.013) compared to patients with low blood eosinophils (Table 3), independent of high BAL eosinophils. Indeed, high BAL eosinophils were only significantly associated with CLAD-free survival (HR = 1.89; CI: 1.24–2.87; *p* = 0.003), but not with graft survival. In the same model, acute rejection, the presence of DSAs and infection at peak blood eosinophilia remained independently associated with CLAD.

## 4. Discussion

In this study, we demonstrated that a high peripheral blood eosinophil percentage (≥8%) is associated with worse outcomes after LTx, i.e., a shorter graft and CLAD-free survival. Strikingly, experiencing multiple episodes of high blood eosinophils did not result in different outcomes; indeed, experiencing only one episode of high blood eosinophils was already associated with a worse outcome. High blood eosinophils ≥8% appeared to be specifically related to the RAS phenotype of CLAD. High BAL eosinophils seemed to be more associated with CLAD, while high blood eosinophils correlated better with survival. However, experiencing both high BAL and high blood eosinophils during follow-up resulted in the worst prognosis.

Increased blood eosinophils is a common feature associated with allergic asthma, where eosinophils are defined as important mediators in its pathophysiology. In response to the recognition of an antigen in the respiratory tract, specific interleukins (IL-4, IL-5 and IL-13) and chemokines (eotaxin) are produced, leading to the attraction of eosinophils to the airways, where they will produce inflammatory mediators that induce inflammation and tissue degeneration [8]. Additionally, eosinophils also play a role in pulmonary fibrosis. Peterson et al. showed that BAL eosinophilia is a negative prognostic marker in patients with idiopathic pulmonary fibrosis [16]. In COPD patients, it was shown that blood eosinophilia was associated with an increased risk of exacerbations, suggesting that elevated levels of blood eosinophils might be a prognostic marker for future COPD exacerbations [17].

Although these studies showed a prominent role of eosinophils in chronic lung diseases, little is known about the role of eosinophils after LTx. Previously, we demonstrated that high BAL eosinophils (≥2%) are correlated with worse outcome post-transplantation, which is confirmed by our present findings [7]. In addition, we demonstrated that patients who experienced both high blood (≥8%) and high BAL eosinophils (≥2%) during their follow-up have a worse prognosis compared to patients who only have high blood or only high BAL eosinophils. According to our multivariate analyses, it seemed that a high BAL eosinophil percentage was more correlated with CLAD-free survival, while high peripheral blood eosinophils were stronger associated with overall survival. This may be the result of peripheral blood reflecting more the general condition of the patient, while BAL reflects the status of the lungs. Therefore, high blood eosinophils may be related to other problems in addition to CLAD, such as pulmonary infections, one of the major complications post-LTx due to the continuous use of immunosuppressives [1]. Indeed, eosinophils are also involved in the host defense and immunologic reaction against respiratory fungal, bacterial and viral pathogens. During infections, eosinophils have multiple functions such as antigen sensing followed by migration to the site of infection. Through their granule content, including proteins and cytokines, eosinophils can induce direct defense strategies to eliminate the pathogens. Furthermore, eosinophils can take part in regulating the immune response by the attraction of other immune cells via stored chemokines [11,18]. Indeed, more patients had an active infection during their peak blood eosinophilia in the high eosinophil group compared to the group with low eosinophils. However, our multivariate analysis showed the association between worse graft survival and high blood eosinophils, independent of active infection during the peak eosinophilia.

Besides the shorter CLAD-free survival in both groups with high blood and high BAL eosinophils, our group previously demonstrated the histological presence of increased eosinophils in explanted end-stage CLAD lungs compared to control lungs [19]. Recently, Darley et al. also found an association between the presence of eosinophils in transbronchial biopsies (TBBs) and an increased risk of CLAD and death [20]. Interestingly, also in this study, a link was found between eosinophils detected in biopsies and the RAS phenotype, although this was not statistically significant. Similarly, our group recently demonstrated the association between late-onset “acute fibrinous and organizing pneumonia” (AFOP) in TBBs and lower CLAD-free and graft survival [21]. Remarkably, late-term AFOP was specifically correlated with increased BAL and blood eosinophilia and the subsequent development of RAS.

This makes us speculate about a possible role for eosinophils in the mechanism of CLAD and more specifically in RAS. Their role could be active, participating in the immune response of the host against donor lung(s), recruiting and activating alloreactive immune cells. Additionally, eosinophils can play a role in tissue remodeling by the release of transforming growth factor-β (TGF-β), which is a known inducer of fibrosis [22]. Previously, we showed the importance of TGF-β in the pathophysiology of RAS via mesothelial-to-mesenchymal transition (MMT) [23]. However, another possibility is that these eosinophils act as immune regulators, trying to keep the immune homeostasis in this complex transplant setting by modulating the immune microenvironment [24]. The exact function of eosinophils and their interplay with other immune cells within transplant rejection remains unclear and therefore deserves more attention.

However, to develop such a complicated disease as CLAD, many more factors than only eosinophils will play a role. Different risk factors for CLAD have been described such as episodes of acute rejection, lymphocytic bronchiolitis and the presence of DSAs, however, the observed effect of high blood eosinophils predicting graft (*p* = 0.036) and CLAD-free survival (*p* = 0.013) proved to be robust in multivariate analysis, adjusting for these established confounding covariates. As meropenem is known to induce peripheral blood eosinophilia [15], this was included in the multivariate analysis, but did not seem to be a confounding factor for high peripheral blood eosinophils.

The therapeutic management of transplant patients with an increased blood eosinophil percentage is not yet clear. However, in 2019 our group showed the efficacy of montelukast post-LTx in patients with an increased blood eosinophil level [25]. Montelukast is a cysteinyl leukotriene receptor antagonist with anti-inflammatory effects, targeting eosinophil chemotaxis among others. Within our LTx cohort, the use of montelukast was associated with a significant attenuation of lung function decline in patients with established CLAD, more specifically BOS. Interestingly, patients who showed a positive response on montelukast after three months had an initial higher blood eosinophil percentage at the start of treatment. Therefore, blood eosinophilia might be a biomarker for response to montelukast in CLAD patients. The exact value of peripheral blood eosinophilia and its management is currently still unknown, but could serve as an additional tool for monitoring the pulmonary graft, as has been postulated previously [26].

We acknowledge some limitations associated with this study, as it is single-centered and has a retrospective design. Furthermore, the etiology of every episode of high blood eosinophils (≥8%) was not investigated in depth. Only peripheral blood eosinophil percentages were used, while lung transplant recipients have common leukopenia and therefore absolute count of eosinophils might be of more interest. However, the incidence of abnormal white blood cell (WBC) count (<4 × 10^9^/L) did not significantly differ between both groups at peak eosinophilia. In addition, the effect of medication and different immunosuppressive agents on the peripheral eosinophil percentages was not examined. Although, all patients received a standardized immunosuppressive regimen, including corticosteroids, and close follow-up treatment.

We conclude that high peripheral blood eosinophils ≥8% is associated with worse graft and CLAD-free survival after LTx independent of high BAL eosinophils, among others. These findings require additional research and may lead to further unraveling of the role of eosinophils after LTx, especially in CLAD.

## Figures and Tables

**Table 1:** Patient characteristics stratified according to high and low peripheral blood eosinophils.

	Low Blood Eosinophils (<8%)	High Blood Eosinophils (≥8%)	*p*-Value
Number of patients, *n* (%)	274 (72.9%)	102 (27.1%)	
Gender female, *n* (%)	122 (44.5%)	64 (62.7%)	**0.002**
Age, years, median (IQR)	57 (47–61)	55 (34.5–61)	0.18
*Native disease, n (%)*			**0.014**
Emphysema. α-1ATD	153 (55.8%)	50 (49%)	
CF/Bronchiectasis	36 (13.2%)	24 (23.5%)	
Pulmonary fibrosis	56 (20.4%)	15 (14.7%)	
PPH	12 (4.4%)	1 (1%)	
Other	17 (6.2%)	12 (11.8%)	
*Type Tx, n (%)*			0.30
SSL	267 (97.4%)	96 (94.1%)	
SSL + liver	3 (1.1%)	3 (2.9%)	
SSL + kidney	1 (0.4%)	0	
HL	3 (1.1%)	3 (2.9%)	
*Outcome, n (%)*			**0.0002**
Alive	236 (86.1%)	69 (67.6%)	
Death	33 (12.1%)	28 (27.5%)	
Retransplantation	5 (1.8%)	5 (4.9%)	
*Cause of death or retransplantation, n (%)*			**0.048**
CLAD	12 (31.6%)	21 (63.6%)	
BOS	7 (58%)	7 (33.4%)	
RAS	5 (42%)	14 (66.6%)	
Infection	11 (28.9%)	3 (9.1%)	
Multifactorial	6 (15.8%)	4 (12.1%)	
Other (e.g., cancer)	9 (23.7%)	5 (15.2%)	
Number of biopsies per patient, median (IQR)	6 (6–7)	7 (6–8)	**0.0047**
Ever AR, *n* (%)	82 (29.9%)	38 (37.3%)	0.21
Ever severe AR (≥A2), *n* (%)	37 (13.5%)	21 (20.6%)	0.11
Mean AR score (St dev)	0.077 (±0.14)	0.11 (±0.21)	0.15
Ever LB, *n* (%)	97 (35.4%)	51 (50%)	**0.012**
Ever severe LB (≥B2), *n* (%)	40 (14.6%)	25 (24.5%)	**0.031**
Mean LB score (St dev)	0.12 (±0.22)	0.17 (±0.24)	**0.008**
Presence of DSAs, *n* (%)	18 (6.6%)	15 (14.7%)	**0.022**
Infection at peak eosinophilia, *n* (%)	69 (25%)	49 (48%)	**<0.0001**
Meropenem at peak eosinophilia, *n* (%)	19 (6.9%)	33 (32.4%)	**<0.0001**
WBC count at peak eosinophilia, median (IQR)	5.58 (4.31–6.89)	6.28 (3.56–7.84)	0.65
Abnormal WBC count at peak eosinophilia (<4 × 10^9^/L), *n* (%)	54 (20%)	29 (28%)	0.09
Time to CLAD, median (IQR)	2.6 years (1.0–4.0)	2.1 years (1.0–3.8)	0.51
Time of follow-up, median (IQR)	4.7 years (3.2–6.6)	4.5 years (3.0–6.5)	0.29

α-1ATD: alpha 1-antitrypsin deficiency; CF: cystic fibrosis; PPH: primary pulmonary hypertension; Tx: transplantation; SSL: sequential single lung transplantation; HL: heart-lung transplantation; CLAD: chronic lung allograft dysfunction; BOS: bronchiolitis obliterans syndrome; RAS: restrictive allograft syndrome; AR: acute rejection; LB: lymphocytic bronchiolitis; DSAs: donor-specific antibodies; WBC: white blood cell. The term “ever” indicates whether or not the patients experienced at least one (severe) AR or LB episode during follow-up.

**Table 2:** Patient proportions in survival analysis.

	Total	CLAD	BOS	RAS
Low blood eosinophils	274 (72.9%)	83 (30.3%)	75 (27.4%)	8 (3%)
High blood eosinophils	102 (27.1%)	62 (60.8%)	38 (37.3%)	24 (23.5%)
Low BAL eosinophils	313 (83.2%)	103 (32.9%)	89 (28.4%)	14 (4.5%)
High BAL eosinophils	63 (16.8%)	42 (66.7%)	24 (38.1%)	18 (28.6%)

CLAD: chronic lung allograft dysfunction; BOS: bronchiolitis obliterans syndrome; RAS: restrictive allograft syndrome; BAL: bronchoalveolar lavage.

**Table 3:** Univariate and multivariate analyses with death and CLAD as primary outcome parameters.

	Univariate Analyses						Multivariate Analyses					
	Death			CLAD			Death			CLAD		
	HR	(CI)	*p*-Value	HR	(CI)	*p*-Value	HR	(CI)	*p*-Value	HR	(CI)	*p*-Value
Age	1.02	(0.99–1.04)	0.1	1.01	(1.00–1.03)	**0.03**	1.02	(0.99–1.05)	0.31	0.99	(0.97–1.01)	0.44
Gender (female)	0.92	(0.58–1.46)	0.72	1.16	(0.83–1.60)	0.39	0.75	(0.46–1.23)	0.26	1.02	(0.72–1.44)	0.92
*Native disease:*												
Emphysema	1.26	(0.78–2.02)	0.34	1.49	(1.06–2.08)	**0.02**	REF					
CF/Bronchiectasis	0.66	(0.32–1.38)	0.27	0.46	(0.26–0.81)	**0.008**	0.82	(0.25–2.69)	0.74	0.26	(0.11–0.64)	0.003
Pulmonary fibrosis	1.19	(0.67–2.10)	0.56	1.14	(0.75–1.72)	0.55	0.84	(0.44–1.59)	0.59	0.7	(0.44–1.01)	0.12
PPH	0.67	(0.16–2.74)	0.58	0.40	(0.13–1.26)	0.12	1.13	(0.23–5.53)	0.88	0.35	(0.09–1.21)	0.09
Other	0.71	(0.26–1.95)	0.51	1.00	(0.55–1.81)	0.99	0.71	(0.21–2.38)	0.58	0.41	(0.19–0.91)	0.03
Any AR	1.44	(0.90–2.31)	0.13	1.51	(1.08–2.10)	**0.016**	1.48	(0.91–2.40)	0.11	1.48	(1.06–2.09)	**0.024**
Any LB	0.69	(0.43–1.13)	0.14	1.00	(0.72–1.39)	0.99	0.74	(0.45–1.24)	0.26	0.99	(0.71–1.40)	0.98
Presence of DSA	2.36	(1.29–4.30)	**0.005**	2.12	(1.33–3.37)	**0.002**	1.63	(0.85–3.13)	0.15	1.82	(1.13–2.91)	**0.013**
Infection at peak blood eosinophils	2.46	(1.54–3.92)	**<0.0001**	1.66	(1.18–2.36)	**0.004**	1.66	(0.92–2.97)	0.09	1.81	(1.21–2.72)	**0.004**
Meropenem at peak blood eosinophils	3.54	(2.15–5.82)	**<0.0001**	1.18	(0.72–1.94)	0.5	1.95	(1.00–3.8)	**0.05**	0.57	(0.32–1.04)	0.065
High BAL eosinophils (≥2%)	1.82	(1.08–3.08)	**0.026**	1.90	(1.28–2.82)	**0.001**	1.36	(0.77–2.4)	0.28	1.89	(1.24–2.87)	0.003
High blood eosinophils (≥8%)	2.44	(1.53–3.89)	**<0.0001**	1.70	(1.19–2.41)	**0.003**	1.81	(1.04–3.16)	**0.036**	1.67	(1.11–2.5)	**0.013**

For the univariate and multivariate analyses with death as primary outcome, the *n* values of graft survival are used: high blood eosinophil group *n* = 102 and high BAL eosinophil group *n* = 63. For the univariate and multivariate analyses with CLAD as primary outcome, the *n* values of CLAD-free survival are used: high blood eosinophil group *n* = 85 and high BAL eosinophil group *n* = 53. CLAD: chronic lung allograft dysfunction; CF: cystic fibrosis; PPH: primary pulmonary hypertension; AR: acute rejection; LB: lymphocytic bronchiolitis; DSA: donor-specific antibodies; BAL: bronchoalveolar lavage.

## Appendix A

**Table A1:** Infection incidence at peak blood eosinophilia.

	Low Blood Eosinophils	High Blood Eosinophils
Infection, *n* (%)	69/274 (25%)	49/102 (48%)
Pulmonary	45 (65.2%)	34 (69.4%)
Bacterial	20 (44.4%)	9 (26.5%)
Viral	12 (26.7%)	6 (17.6%)
Fungal	6 (13.3%)	7 (20.6%)
Multiple pathogens	7 (15.6%)	12 (35.3%)
Extrapulmonary	7 (10.2%)	4 (8.2%)
Unknown	17 (24.6%)	11 (22.4%)
